# Carbohydrate-binding modules enhance H_2_O_2_ tolerance by promoting lytic polysaccharide monooxygenase active site H_2_O_2_ consumption

**DOI:** 10.1016/j.jbc.2023.105573

**Published:** 2023-12-18

**Authors:** Wa Gao, Tang Li, Haichuan Zhou, Jiu Ju, Heng Yin

**Affiliations:** 1 Dalian Engineering Research Center for Carbohydrate Agricultural Preparations, Dalian Technology Innovation Center for Green Agriculture, Liaoning Provincial Key Laboratory of Carbohydrates, Dalian Institute of Chemical Physics, Chinese Academy of Sciences, Dalian, China; 2 University of Chinese Academy of Sciences, Beijing, China

**Keywords:** lytic polysaccharide monooxygenase, carbohydrate-binding module, cellulose, substrate binding affinity, activity, H_2_O_2_ tolerance

## Abstract

Lytic polysaccharide monooxygenases (LPMOs) oxidatively depolymerize recalcitrant polysaccharides, which is important for biomass conversion. The catalytic domains of many LPMOs are linked to carbohydrate-binding modules (CBMs) through flexible linkers, but the function of these CBMs in LPMO catalysis is not well understood. In this study, we utilized *Mt*LPMO9L and *Mt*LPMO9G derived from *Myceliophthora thermophila* to investigate the impact of CBMs on LPMO activity, with particular emphasis on their influence on H_2_O_2_ tolerance. Using truncated forms of *Mt*LPMO9G generated by removing the CBM, we found reduced substrate binding affinity and enzymatic activity. Conversely, when the CBM was fused to the C terminus of the single-domain *Mt*LPMO9L to create *Mt*LPMO9L-CBM, we observed a substantial improvement in substrate binding affinity, enzymatic activity, and notably, H_2_O_2_ tolerance. Furthermore, molecular dynamics simulations confirmed that the CBM fusion enhances the proximity of the active site to the substrate, thereby promoting multilocal cleavage and impacting the exposure of the copper active site to H_2_O_2_. Importantly, the fusion of CBM resulted in more efficient consumption of H_2_O_2_ by LPMO, leading to improved enzymatic activity and reduced auto-oxidative damage of the copper active center.

The annual yield of plant biomass contains vast amounts of carbohydrate biopolymers, such as cellulose in plant cell walls (1), making it a valuable feedstock for producing biofuels and green chemicals through degradation (2). Enzymatic catalysis is a promising approach due to its mild reaction conditions, high selectivity, and environmental sustainability (3). Carbohydrate-active enzymes are categorized in the CAZY database (http://www.cazy.org/), including glycoside hydrolases, polysaccharide lyases, and auxiliary activities (AA), along with their appended noncatalytic carbohydrate-binding modules (CBMs) (4). CBMs are contiguous amino acid sequences that possess a discrete fold (5) and carbohydrate-binding property (6, 7). Although some CBMs exist independently, the majority are attached to the catalytic domains (CDs) of carbohydrate-active enzymes *via* peptide linkers (8, 9). CBMs play multiple roles in carbohydrate-active enzymes (10), including enhancing activity (11, 12), improving substrate specificity (13), and enhancing thermostability (14, 15, 16). At present, two common strategies are employed in CBM engineering (17). One involves optimizing the CBM of the enzyme itself *via* point mutation (18, 19), while the other entails fusing additional CBMs to the enzyme (20). Despite the predominant focus on glycoside hydrolase enzymes in CBM engineering, limited information is available regarding other carbohydrate-active enzymes.

In recent years, a novel carbohydrate-active enzyme known as lytic polysaccharide monooxygenase (LPMO) has been discovered (21, 22). LPMOs are categorized under the AA category in the CAZY database, with the the cellulose-active AA9 LPMO being the most extensively studied (23, 24). These enzymes are monocopper enzymes that catalyze the oxidative cleavage of glycosidic bonds in the crystalline region of carbohydrate biopolymers, rendering them more accessible to other glycolytic enzymes (25). The oxidative cleavage of polysaccharide substrates by LPMOs involves the presence of the divalent copper ion in the active site, oxidized cosubstrate (H_2_O_2_ or O_2_), and reductant, working in concert to oxidize the glycosidic bond. This process results in oxidized C1-(lactone) or C4-(ketoaldose) positions or a mixture of both (26). Recent studies have demonstrated that H_2_O_2_ serves as a more efficient cosubstrate for LPMO than O_2_ (27, 28, 29). However, it is important to note that H_2_O_2_ may cause autocatalytic damage to copper active centers, especially at low substrate concentrations (29). Hence, enhancing H_2_O_2_ tolerance is crucial for maintaining enzymatic activity, which has significant potential for the industrial applications of LPMO.

Similar to other CAZymes, a significant proportion of LPMOs (approximately 30%) contain CBMs (3). However, only a few studies have delved into the role of CBMs in LPMOs, leading to an undercharacterized understanding of their impact on substrate binding and enzymatic activity. The removal of CBMs from two AA10 LPMOs caused a modest reduction in activity against phosphate-swollen cellulose (PASC) (22) and Avicel (30), respectively. Other studies have suggested that CBM2a and CBM3a modules affect the activity and substrate specificity of AA10 LPMOs and may even modulate their action mode (31). CBMs may also play a crucial role in the stability of LPMOs, as their proximity to the substrate not only enhances enzyme efficiency but also shields the enzyme from autocatalytic inactivation (32). Recent research has highlighted the significance of the binding affinity of CBM-containing LPMOs in relation to the efficacy of H_2_O_2_ as a cosubstrate (2). However, the effect of CBMs on the *in situ* generation and consumption of H_2_O_2_ requires further investigation. Therefore, a more comprehensive analysis of the role of CBMs in LPMO catalysis is necessary.

In our previous research, we demonstrated that dual-domain *Mt*LPMO9G (GenBank code: MYCTH-110651) and single-domain *Mt*LPMO9L (GenBank code: MYCTH-103537) from *Myceliophthora thermophile* can cleave cellulose by oxidizing C1 in the β-1,4-glycosidic bond (33, 34, 35). Through a combination of *in silico* and biochemical methods, we uncovered a correlation between the accumulation of H_2_O_2_ in enzymatic reactions and the substrate binding capacity of LPMOs. Notably, LPMOs engaged in the productive binding of insoluble polysaccharides not only failed to accumulate H_2_O_2_ but also actively consumed it. In this study, we conducted CBM truncation and fusion experiments employing *Mt*LPMO9G and *Mt*LPMO9L as model enzymes to elucidate the role of the CBM. We enhanced the substrate binding affinity, activity, and H_2_O_2_ tolerance of LPMO through CBM engineering. Furthermore, we assessed the impact of CBM on the accessibility of H_2_O_2_ to the copper active sites in LPMO using molecular dynamics (MD) simulations.

## Results

### Removal of the non-CD in *Mt*LPMO9G and expression of truncated variants

**Figure 1:**
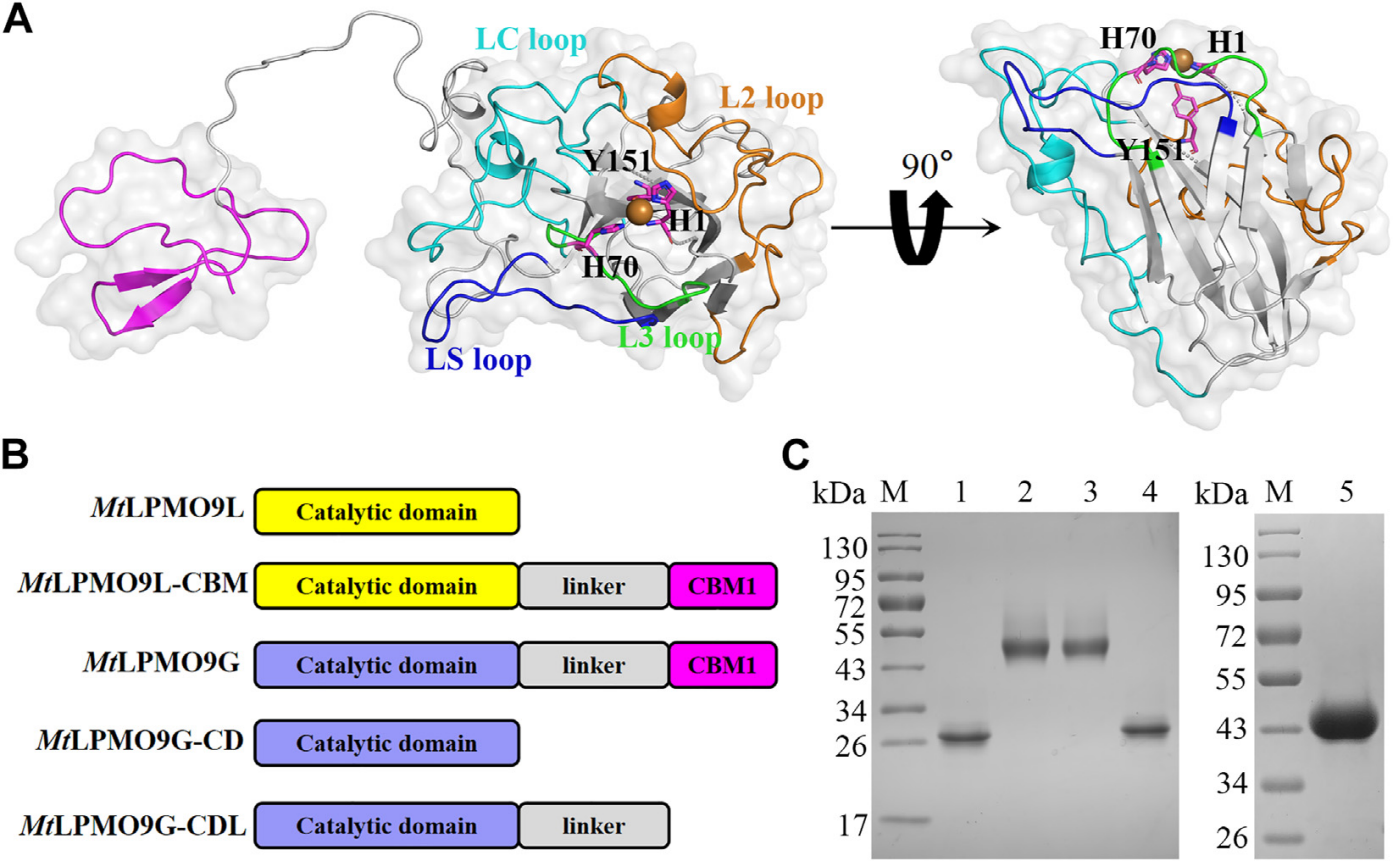
**Structural modeling of *Mt*LPMO9G and *Mt*LPMO9G-CD, as well as design and purification of CBM-engineered variants.***A*, Rosettafold-generated three-dimensional structures of *Mt*LPMO9G and *Mt*LPMO9G-CD. The substrate binding surface of the catalytic domain consists of five loops marked with different colors (33, 34). *B*, schematic representation of the enzyme modularization strategy for CBM-engineered variants. *C*, SDS-PAGE analysis of purified *Mt*LPMO9G, *Mt*LPMO9L, and both variants. *Lane M* represents the molecular weight marker, and lanes 1 to 5 represent purified *Mt*LPMO9L, *Mt*LPMO9L-CBM, *Mt*LPMO9G, *Mt*LPMO9G-CD, and *Mt*LPMO9G-CDL, respectively. CBM, carbohydrate-binding module; CD, catalytic domain; LPMO, lytic polysaccharide monooxygenase.

The dual-domain enzyme *Mt*LPMO9G consists of a catalytic AA9 domain and a CBM1 domain attached by a linker, comprising a total of 284 amino acids (excluding signal peptide) (Fig. 1*B*). To investigate the role of CBM in *Mt*LPMO9G, we generated truncated variants of the enzyme. The first variant *Mt*LPMO9G-CDL was created by removing only the CBM, thus retaining the catalytic domain and the linker. The second variant, *Mt*LPMO9G-CD was produced by eliminating both the CBM and the linker, leaving only the catalytic domain (Fig. 1*B*). *Mt*LPMO9G and its truncated variants were recombinantly expressed in *Pichia pastoris*. The purified proteins obtained through ion exchange chromatography were confirmed by SDS-PAGE analysis (Fig. 1*C*). To obtain a structural impression of *Mt*LPMO9G, we used RoseTTAFold to generate the three-dimensional structures of *Mt*LPMO9G and *Mt*LPMO9G-CD, respectively. The simulated structure reveals that the crucial β-strands assemble into a slightly distorted fibronectin-like/immunoglobulin-like β-sandwich core structure within the CD of *Mt*LPMO9G. The copper active center of *Mt*LPMO9G is characterized by a histidine brace, consisting of two conserved histidine residues (H1 and H70) (Fig. 1*A*).

### Impact of CBM on the binding affinity and activity of *Mt*LPMO9G

**Figure 2:**
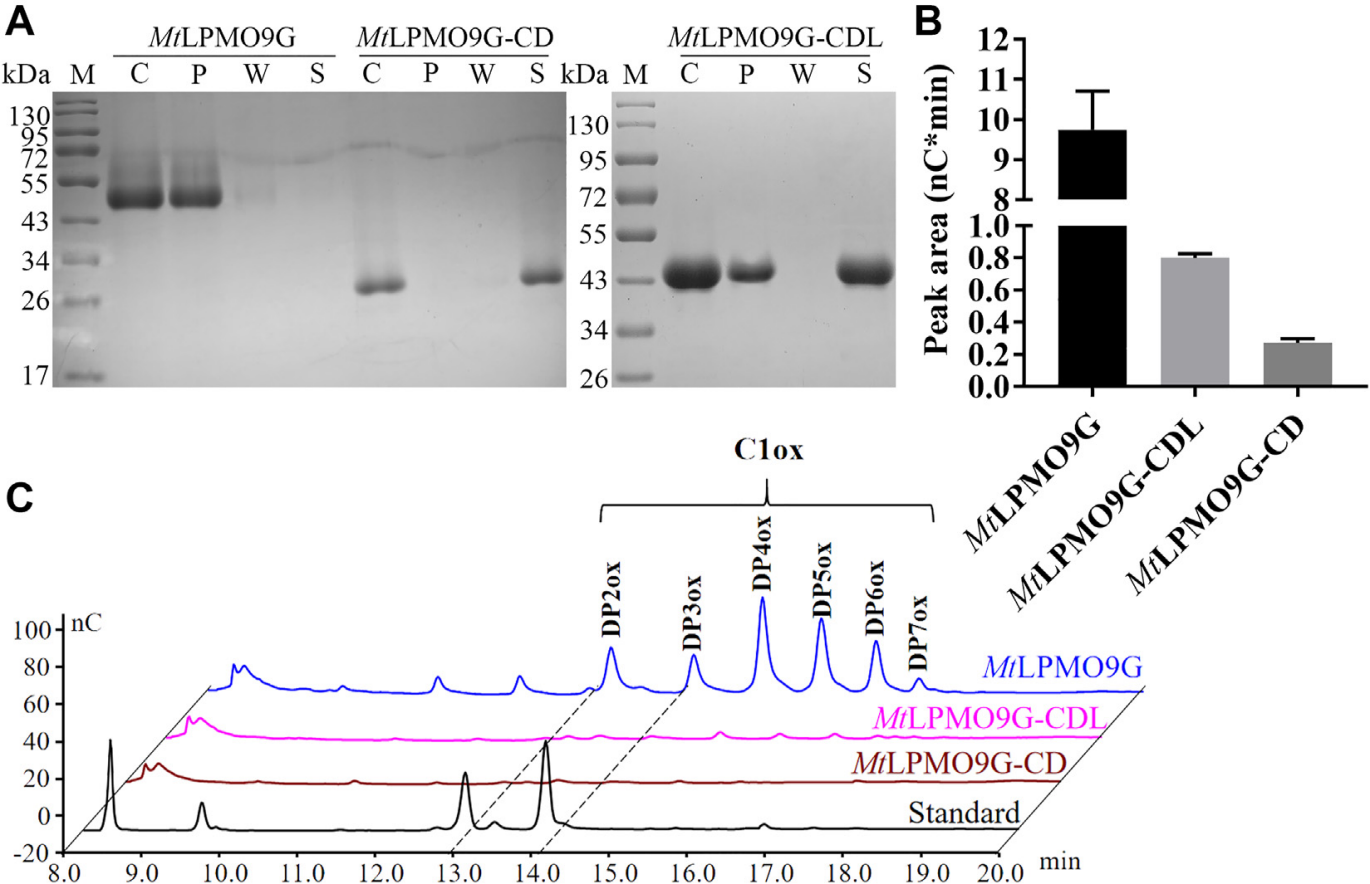
**Substrate binding and activity of *Mt*LPMO9G and its truncated forms**. *A*, substrate binding affinity of *Mt*LPMO9G, *Mt*LPMO9G-CDL, and *Mt*LPMO9G-CD compared by affinity precipitation. *Lane M* represents the molecular weight marker; *Lane C* represents the enzyme without substrate under the same conditions as a control group; *Lanes P, W,* and *S* represent precipitation, washing solution, and supernatant, respectively. *B*, activity quantification of *Mt*LPMO9G, *Mt*LPMO9G-CDL, and *Mt*LPMO9G-CD. *C*, the activity of *Mt*LPMO9G, *Mt*LPMO9G-CDL, and *Mt*LPMO9G-CD on PASC compared by HPAEC. CBM, carbohydrate-binding module; CD, catalytic domain; HPAEC, high performance anion exchange chromatography; PASC, phosphate-swollen cellulose; LPMO, lytic polysaccharide monooxygenase.

We compared the substrate binding affinity of *Mt*LPMO9G, *Mt*LPMO9G-CDL, and *Mt*LPMO9G-CD to PASC by performing affinity precipitation assays. Each LPMO was individually incubated with PASC for 24 h in the absence of ascorbic acid (AscA) to prevent any reaction between the enzyme and the substrate. As depicted in Figure 2*A*, the protein band of *Mt*LPMO9G was predominantly present in the precipitant lane, indicating a robust interaction with the substrate. In contrast, the protein band of *Mt*LPMO9G-CD was mainly detected in the supernatant lane, suggesting a reduced substrate binding affinity compared to the dual-domain *Mt*LPMO9G containing the CBM. Notably, in the case of *Mt*LPMO9G-CDL, most of the protein band was found in the supernatant lane, indicating a diminished substrate binding affinity compared to *Mt*LPMO9G. However, a small portion of the protein band remained in the precipitant lane, suggesting that the linker unexpectedly retains some substrate binding affinity. Although linkers are generally considered as flexible spacers between two domains, some studies have demonstrated that linkers can contribute to polysaccharide binding (36), aligning with our observed results.

Furthermore, we compared the activity of *Mt*LPMO9G and its truncated forms on PASC by high-performance anion exchange chromatography (HPAEC). As shown in Figure 2*C*, the products released from PASC consisted of a series of oxidized cello-oligosaccharides with various degrees of polymerization from DP2 to DP7. Both *Mt*LPMO9G and its truncated forms produced only C1-oxidized cello-oligosaccharides, indicating that removal of CBM has no effect on the oxidation regioselectivity. To further assess enzymatic activity, we quantified the products by HPAEC (Fig. 2*B*). The presence of the linker resulted in a marginal increase in the activity of *Mt*LPMO9G-CDL compared to *Mt*LPMO9G-CD. However, the product yield of *Mt*LPMO9G exceeded that of both truncated forms by more than 10-fold, suggesting that the removal of CBM from *Mt*LPMO9G dramatically decreased the activity on cellulose. This decline in activity can be attributed to the reduced substrate binding affinity and enzyme concentration on the substrate surface, resulting from the loss of CBM (2).

### Fusion of CBM and expression of *Mt*LPMO9L-CBM

**Figure 3:**
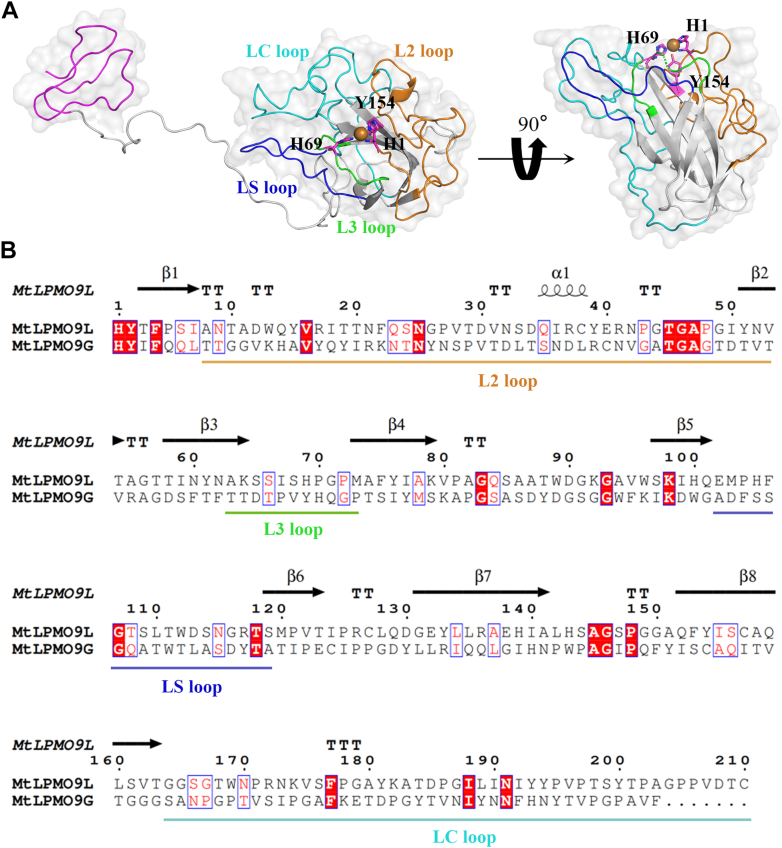
**Structural modeling of *Mt*LPMO9L and *Mt*LPMO9L-CBM, and sequence alignment between *Mt*LPMO9L and *Mt*LPMO9G.***A*, Rosettafold-generated three-dimensional structures of *Mt*LPMO9L and *Mt*LPMO9L-CBM. The substrate binding surface of the catalytic domain consists of five loops marked with different colors (33, 34). *B*, sequence alignment of *Mt*LPMO9L and *Mt*LPMO9G. Multiple-sequence alignment was performed using ClustalW software (http://www.genome.jp/tools-bin/clustalw), and the results were plotted using ESPript 3.0 (http://espript.ibcp.fr/ESPript/cgi-bin/ESPript.cgi) for better visualization. CBM, carbohydrate-binding module; LPMO, lytic polysaccharide monooxygenase.

*Mt*LPMO9L is a single-domain enzyme consisting of 210 amino acids (excluding signal peptide) (Fig. 1*B*), with 30% sequence similarity to *Mt*LPMO9G (Fig. 3*B*). To further probe the function of CBM, we fused the non-CD module (AA206–AA284) of *Mt*LPMO9G to the C terminus of *Mt*LPMO9L, creating the fused form *Mt*LPMO9L-CBM. Both *Mt*LPMO9L and *Mt*LPMO9L-CBM were recombinantly expressed in *P. pastoris*. The purified proteins obtained through ion exchange chromatography were confirmed by SDS-PAGE analysis (Fig. 1*C*). Utilizing RoseTTAFold, we generated three-dimensional structures for *Mt*LPMO9L and *Mt*LPMO9L-CBM, revealing that key β-strands form a slightly twisted β-sandwich structure within the CD. The histidine brace of *Mt*LPMO9L and *Mt*LPMO9L-CBM consists of two conserved histidine residues (H1 and H69) located at the copper active center (Fig. 3*A*) (33, 34).

### Fusion of CBM enhances substrate binding affinity and enzymatic activity of *Mt*LPMO9L-CBM

**Figure 4:**
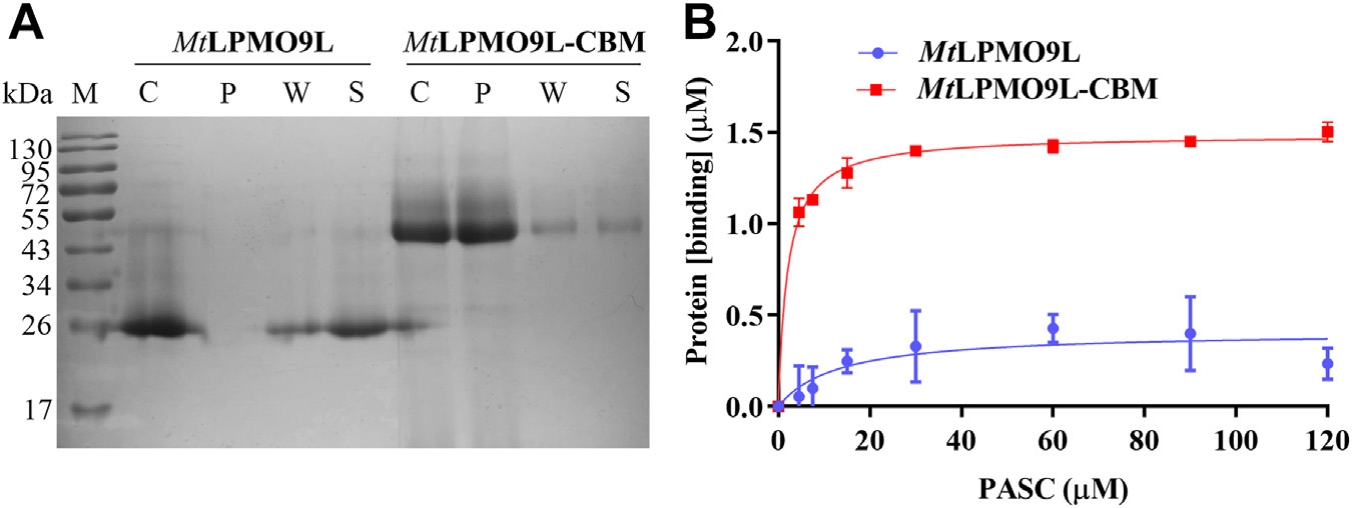
**Comparison of substrate binding affinity between *Mt*LPMO9L and *Mt*LPMO9L-CBM.***A*, substrate binding affinity of *Mt*LPMO9L and *Mt*LPMO9L-CBM compared by affinity precipitation. *Lane M* represents the molecular weight marker; *Lane C* represents the enzyme without substrate under the same conditions as a control group; *Lanes P, W,* and *S* represent precipitation, washing solution, and supernatant, respectively. *B*, substrate binding affinity of *Mt*LPMO9L and *Mt*LPMO9L-CBM compared by pull-down assay. The data is fitted to a Langmuir adsorption curve. CBM, carbohydrate-binding module; LPMO, lytic polysaccharide monooxygenase.

To compare the substrate binding affinities of *Mt*LPMO9L and *Mt*LPMO9L-CBM to PASC, we conducted affinity precipitation and pull-down assays. The affinity precipitation results (Fig. 4*A*) showed that the *Mt*LPMO9L-CBM protein band primarily appeared in the precipitant lane, indicating a robust binding affinity to the substrate. In contrast, the *Mt*LPMO9L protein band mainly appeared in the supernatant lane, with a small amount in the washing solution lane, indicating weaker binding to the substrate. The pull-down assays were performed using saturation binding experiments (33) and fitted with the Langmuir adsorption isotherm (Fig. 4*B*), as previously reported by Hansson *et al*. (3). The *K*_*d*_ and maximum binding capacity (*B*_max_) of *Mt*LPMO9L and *Mt*LPMO9L-CBM are listed in Table S3. These results demonstrated that the fusion of CBM significantly enhanced the substrate binding affinity of *Mt*LPMO9L-CBM by at least 3-fold compared to that of *Mt*LPMO9L alone, confirming the important role of the CBM domain in the substrate binding of *Mt*LPMO9L-CBM.

**Figure 5:**
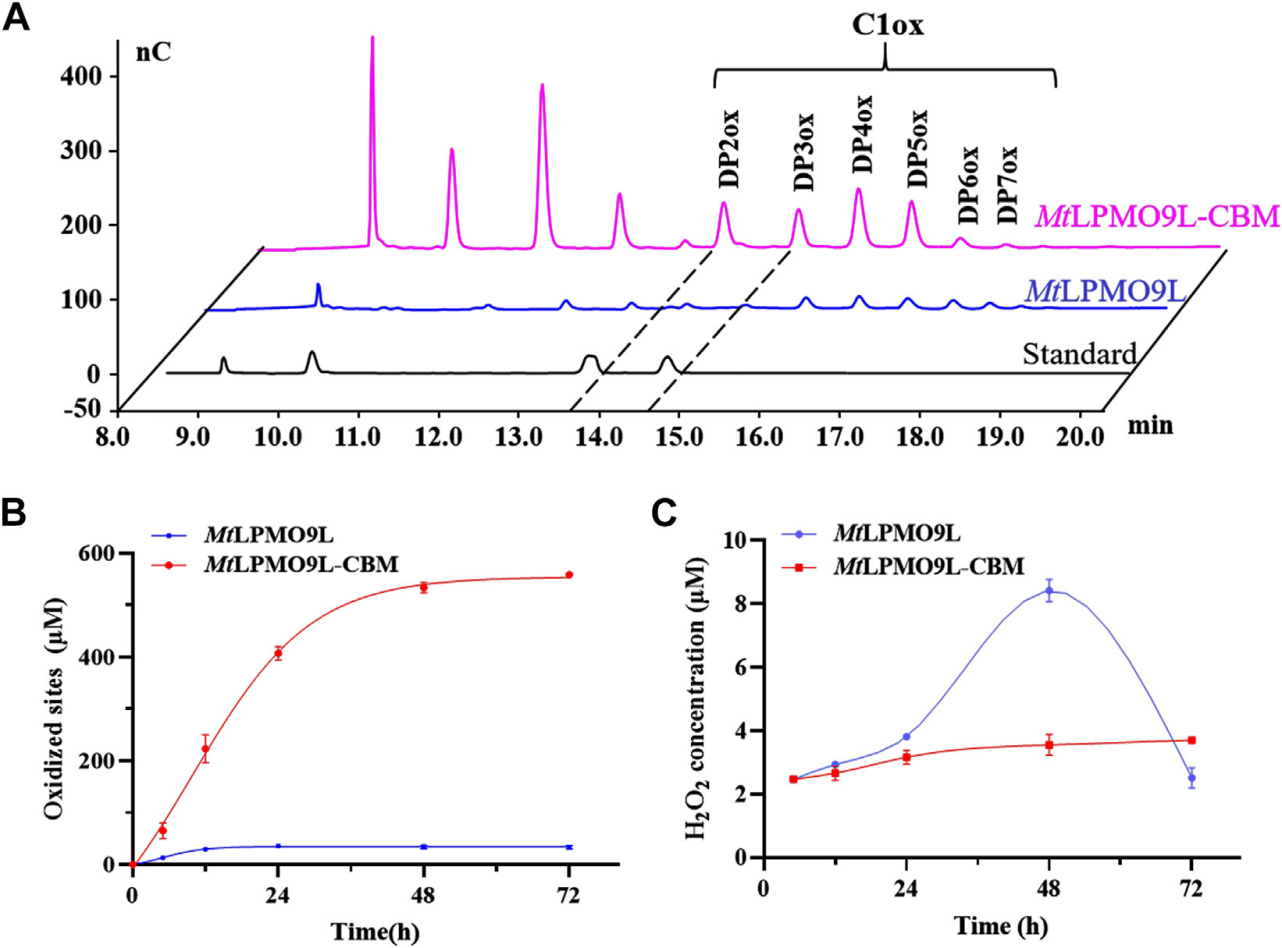
**Enzymatic activity of *Mt*LPMO9L and *Mt*LPMO9L-CBM**. *A*, analysis of the activity of *Mt*LPMO9L and *Mt*LPMO9L-CBM on PASC by HPAEC. *B*, time-course experiment comparing the activity of *Mt*LPMO9L and *Mt*LPMO9L-CBM on PASC. The concentration of PASC was 4 mg mL^−1^, and the products were quantitatively analyzed by HPAEC. *C*, H_2_O_2_ content measurement during cellulose degradation by *Mt*LPMO9L and *Mt*LPMO9L-CBM. The measured H_2_O_2_ content represents the net concentration. CBM, carbohydrate-binding module; HPAEC, high performance anion exchange chromatography; LPMO, lytic polysaccharide monooxygenase; PASC, phosphate-swollen cellulose.

To evaluate the effect of CBM on activity, we subsequently compared the activities of *Mt*LPMO9L and *Mt*LPMO9L-CBM on PASC by HPAEC. The results (Fig. 5*A*) showed that both enzymes produced a series of C1-oxidized cello-oligosaccharides with varying degrees of polymerization from DP2 to DP7, indicating that adding CBM to *Mt*LPMO9L did not affect the oxidation regioselectivity. Notably, the product yield of *Mt*LPMO9L-CBM exceeded that of *Mt*LPMO9L, indicating a higher cellulose-degrading activity in the former.

**Figure 6:**
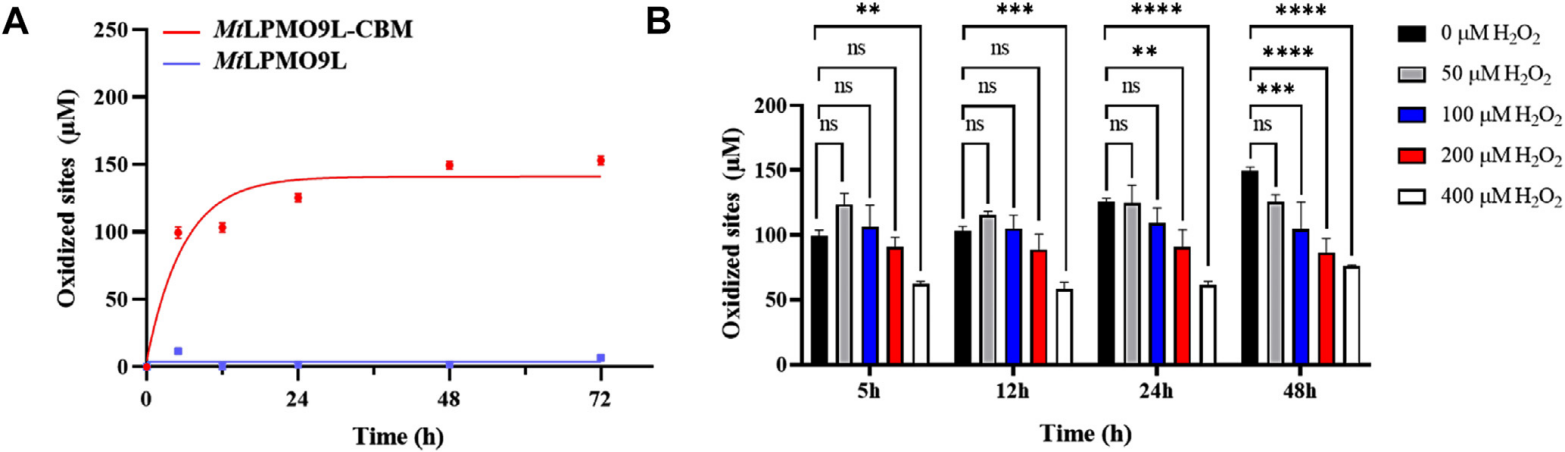
**The activity of *Mt*LPMO9L and *Mt*LPMO9L-CBM on PASC.***A*, time-course experiment measuring the activity of *Mt*LPMO9L and *Mt*LPMO9L-CBM on PASC at a concentration of 1 mg mL^−1^. *B*, H_2_O_2_ tolerance of *Mt*LPMO9L and *Mt*LPMO9L-CBM compared by adding different concentrations of H_2_O_2_ (25, 50, 100, and 200 μM) at the beginning of the reaction with 1 mg mL^−1^ PASC. The oxidized products were quantified by HPAEC. Statistical significance was tested by one-way ANOVA test to compare the H_2_O_2_-added group with the group without H_2_O_2_. Asterisks indicate significant differences (∗∗∗∗*p* ＜ 0.0001, ∗∗∗*p* ＜ 0.001, ∗∗*p* ＜ 0.01, ∗*p* ＜ 0.05, ns *p* ＞ 0.05). CBM, carbohydrate-binding module; HPAEC, high performance anion exchange chromatography; LPMO, lytic polysaccharide monooxygenase; PASC, phosphate-swollen cellulose.

Subsequently, we conducted a time-course analysis of the degradation of 4 mg mL^-1^ PASC under conditions typically employed for LPMO characterization, with the presence of O_2_ and 1 mM AscA. We utilized HPAEC to assess the total oxidation of cellulose by determining the total number of oxidized sites after LPMO catalysis (37). As shown in Figure 5*B*, the concentration of oxidized products in *Mt*LPMO9L-CBM increased in a time-dependent manner, reaching an accumulated amount of 558.51 ± 3.36 μM after 72 h of reaction. In contrast, *Mt*LPMO9L exhibited a gradual accumulation of oxidized products within the first 24 h of the reaction, with no significant accumulation observed in the subsequent 48 h. The final accumulated amount of oxidized products in *Mt*LPMO9L after 72 h of reaction was 33.31 ± 3.34 μM. To further investigate the potential impact of substrate loading on LPMO efficacy, we conducted a time-course analysis using *Mt*LPMO9L and *Mt*LPMO9L-CBM with 1 mg mL^-1^ PASC. Notably, *Mt*LPMO9L-CBM exhibited commendable performance at low substrate concentration, while *Mt*LPMO9L showed minimal detection of oxidized products (Fig. 6*A*). It can reasonably be inferred that the beneficial substrate affinity mediated by CBM facilitates productive binding of LPMO to the substrate (37). Through differential scanning calorimetry (DSC) analysis, we determined that the fusion of CBM did not significantly affect the thermal stability of LPMO (Fig. S1). This implies that there might be other hidden factors influencing operational stability in this context (38).

**Figure 7:**
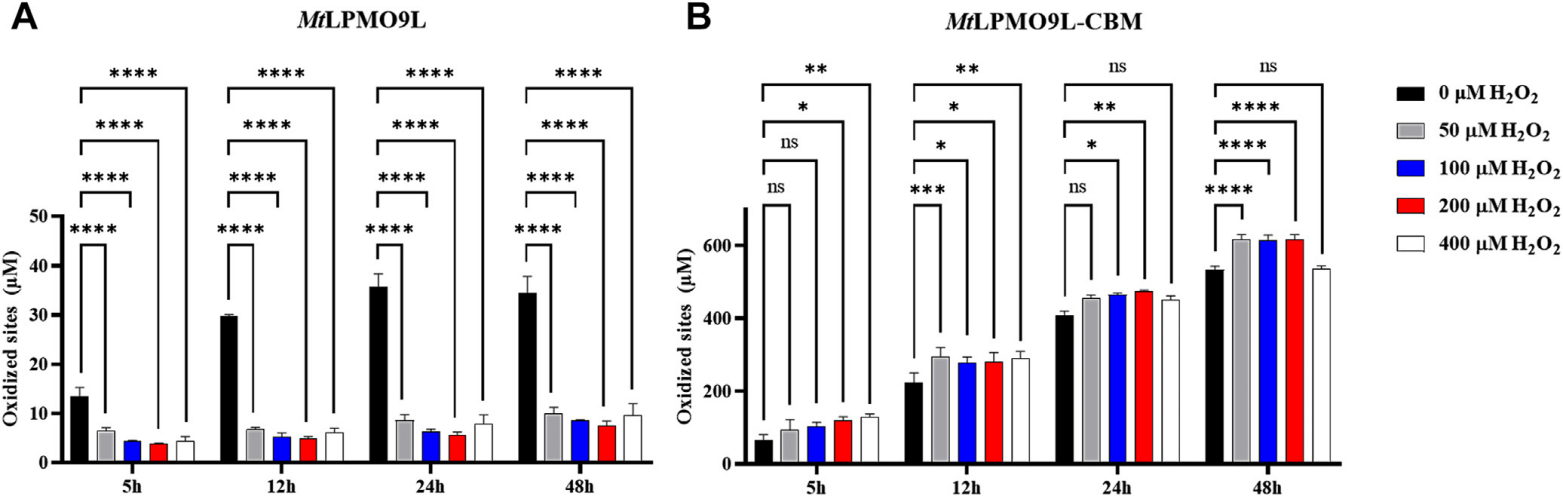
**H**_**2**_**O**_**2**_**tolerance of *Mt*LPMO9L and *Mt*LPMO9L-CBM.***A* and *B*, respectively show the effects of different concentrations of H_2_O_2_ (0, 50, 100, 200, and 400 μM) on the activity of *Mt*LPMO9L and *Mt*LPMO9L-CBM, with H_2_O_2_ added at the beginning of the reaction. The substrate used was 4 mg mL^−1^ PASC, and the oxidized products were quantified by HPAEC. Statistical significance was tested by one-way ANOVA test to compare the H_2_O_2_-added group with the group without H_2_O_2_. *Asterisks* indicate significant differences (∗∗∗∗*p* ＜ 0.0001, ∗∗∗*p* ＜ 0.001, ∗∗*p* ＜ 0.01, ∗*p* ＜ 0.05, ns *p* ＞ 0.05). CBM, carbohydrate-binding module; HPAEC, high performance anion exchange chromatography; LPMO, lytic polysaccharide monooxygenase; PASC, phosphate-swollen cellulose.

We further examined the changes in H_2_O_2_ content in the *Mt*LPMO9L and *Mt*LPMO9L-CBM reaction systems over 72 h. It is noteworthy that LPMO itself can both produce and consume H_2_O_2_ during catalysis process (39). Therefore, only the H_2_O_2_ net concentration was measured in the LPMO reactions. As shown in Figure 5*C*, the H_2_O_2_ net concentration in both *Mt*LPMO9L and *Mt*LPMO9L-CBM reaction systems increased continuously at a similar rate during the first 24 h of the reaction. Subsequently, the H_2_O_2_ net concentration in the *Mt*LPMO9L-CBM system remained relatively stable at 3.71 ± 0.06 μM. In contrast, the H_2_O_2_ net concentration in the *Mt*LPMO9L system reached a maximum of 8.42 ± 0.29 μM after 48 h of incubation, followed by a gradual decline to 2.52 ± 0.26 μM in the last 24 h. An appropriate concentration of H_2_O_2_ is critical for maintaining the optimal activity of LPMOs, ensuring a sufficient supply of the oxidant while avoiding damage to the copper active center (38). While *Mt*LPMO9L retained enzymatic activity during the initial 24 h, the rapid accumulation of H_2_O_2_ and the almost negligible accumulation of oxidized products in the subsequent 24 h suggest that, during this phase, *Mt*LPMO9L primarily undergoes a reductant oxidase reaction rather than oxidative degradation of substrate (40). This ultimately led to complete inactivation of *Mt*LPMO9L after 48 h, with no further increase in H_2_O_2_ content, and even a gradual decrease due to factors such as reductants and environmental conditions in the system. These results indicated that *Mt*LPMO9L-CBM may have a superior H_2_O_2_ utilization efficiency than *Mt*LPMO9L, as the product yield was higher and the H_2_O_2_ net concentration was lower in the system.

In most typical reaction setups, LPMO activity is constrained by the *in situ* generation of H_2_O_2_. The CBM not only enhances the operational stability of LPMOs under turnover conditions but also effectively prevents LPMO's auto-oxidative inactivation (41). On one hand, the CBM aids in bringing the CD of LPMO closer to the substrate, increasing the chances of effective H_2_O_2_ utilization (33). On the other hand, substrate binding impedes the oxidative enzyme activity of LPMO.

### Fusion of CBM1 substantially enhanced the H_2_O_2_ tolerance of *Mt*LPMO9L-CBM

To further validate the role of CBM in mitigating the constraints of H_2_O_2_ levels on LPMO activity, we introduced varying concentrations of H_2_O_2_ (0, 50, 100, 200, and 400 μM) at the outset of *Mt*LPM9L and *Mt*LPM9L-CBM reactions, based on 4 mg mL^-1^ PASC. The activity of *Mt*LPMO9L was apparently inhibited at all concentrations of H_2_O_2_ (Fig. 7*A*), while *Mt*LPMO9L-CBM consistently exhibited robust operational stability at all H_2_O_2_ concentrations. As shown in Figure 7*B*, the yield of oxidized products increased over time at all H_2_O_2_ concentrations, indicating the commendable activity of *Mt*LPMO9L-CBM. We further reduced the substrate concentration of PASC from 4 mg mL^-1^ to 1 mg mL^-1^ and monitored the formation of oxidized products over time (Fig. 6*B*). As expected, no oxidized products were detected for *Mt*LPMO9L at all H_2_O_2_ concentrations (not shown in the figure), indicating that *Mt*LPMO9L rapidly deactivated at the beginning of the reaction. In contrast, the presence of 50 μM H_2_O_2_ had a promoting effect on the accumulation of oxidized products by *Mt*LPMO9L-CBM in the initial 12 h of the reaction. Although the enzyme activity of *Mt*LPMO9L-CBM moderately decreased in subsequent time intervals and at other H_2_O_2_ concentrations, it is undeniable that the tolerance of *Mt*LPMO9L-CBM to H_2_O_2_ remained satisfactory compared to *Mt*LPMO9L at a substrate concentration of 1 mg mL^-1^ PASC.

As Crouch *et al*. pointed out, CBMs can guide and modify the activity of LPMOs (31). Promoting binding to internal sites on the substrate surface is one way of CBM to enhance enzymatic activity, as it can facilitate multiple cleavages in the same region (42). Furthermore, this tight binding effectively protects the copper active center from nonproductive oxidative damage by H_2_O_2_ (2).

### MD simulations illustrate the essential role of CBM1 for LPMO

As widely acknowledged, H_2_O_2_ serves as an important cosubstrate for LPMO activity. However, excess H_2_O_2_ can hamper the activity of LPMO through auto-oxidative inactivation of copper active centers. The above-mentioned results indicate that the fusion of CBM improves the H_2_O_2_ tolerance of *Mt*LPMO9L-CBM. To further investigate the role of CBM on the interaction between the copper ion active center and H_2_O_2_, we performed MD simulations based on these three systems: *Mt*LPMO9L-cellulose-H_2_O_2_, *Mt*LPMO9L-CBM-cellulose-H_2_O_2_, and *Mt*LPMO9L-CBM-H_2_O_2_. The initial positions of the CD on the cellulose surface were established using a similar method as previously described (33). As for the CBM, it belongs to the CBM1 category in the CAZy database (http://www.cazy.org/CBM1.html) and shares homology with *Tr*Cel7A's CBM. When the CBM of *Mt*LPMO9G was overlaid with *Tr*Cel7A's CBM (Fig. S2), it was observed that residues Trp258, Asn282, Tyr284, Tyr285, and Gln287 (corresponding to Try5, Asn29, Tyr31, Tyr32, and Gln34 in *Tr*Cel7A) were involved in binding to the cellulose surface (43). Therefore, by manually placing the modules on the cellulose surface, the interacting residues (Trp258, Asn282, Tyr284, Tyr285, and Gln287) were oriented toward the surface and maintained a minimal distance within a certain range, thus defining the starting position of the CBM on the cellulose surface. Additionally, the positions of all H_2_O_2_ molecules in all systems were randomly placed.

**Figure 8:**
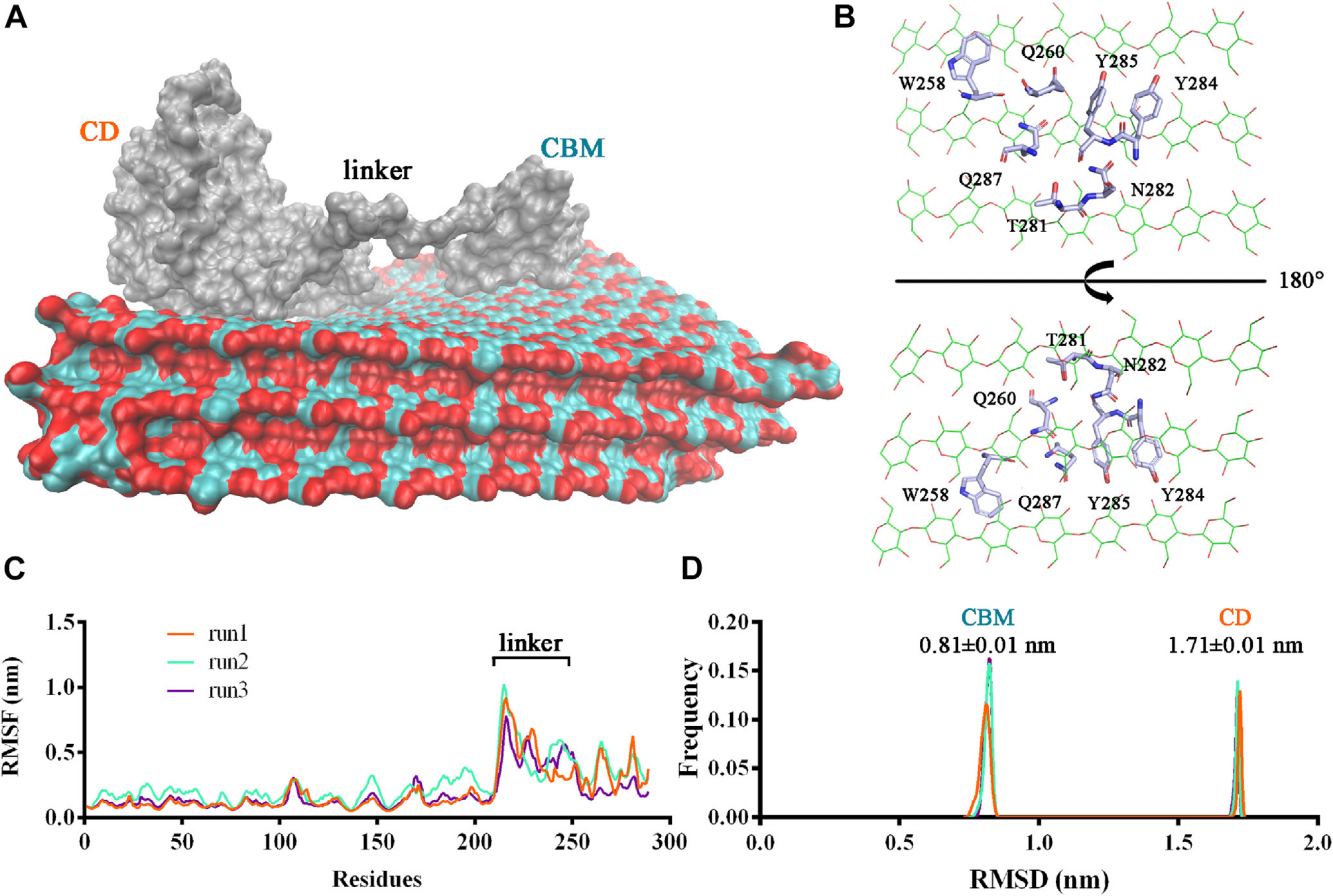
**Interactions of *Mt*LPMO9L-CBM with cellulose probed by MD simulations.***A*, typical presentation of the overall interaction of *Mt*LPMO9L-CBM with cellulose from MD simulations. *B*, close-up view of the interacting surface of CBM and cellulose. Key interacting residues are labeled. *C*, RMSF of the *Mt*LPMO9L-CBM structure (run1, run2, run3). The linker region is labeled. *D*, the frequency distribution of RMSD values of the CD and CBM in all 5001 frames of the MD trajectory (run1, run2, run3). The SD is calculated based on the distribution of RMSD values. The mean RMSD and SD of the CD and CBM from the three independent MD runs are labeled on their corresponding frequency distribution peaks. CBM, carbohydrate-binding module; CD, catalytic domain; LPMO, lytic polysaccharide monooxygenase; MD, molecular dynamics; RMSF RMS fluctuation.

Short-range nonbonded interaction energies were calculated, including van der Waals’ forces modeled by Lennard-Jones short-range potential and Columbic short-range electrostatic. The results showed that the CBM exhibited a comparable nonbonded interaction energy to the cellulose (averaging 275.03 ± 0.96 kJ mol^−1^ from the three independent runs), in contrast to the CD (averaging 270.14 ± 53.45 kJ mol^−1^ from the three independent runs) in the *Mt*LPMO9L–CBM–cellulose complex system. It indicated that the introduced CBM provided additional binding sites for the enzyme to anchor onto the cellulose surface. In addition, it is obvious that the interaction between cellulose and the two modules (CD and CBM) mainly attribute to the Lennard-Jones short-range potential (Table 1). Within the CBM, residues such as W258, Q260, T281, N282, Y284, Y285, and Q287 engage in the interaction with the cellulose surface through hydrogen bond interactions and alkyl-pi stacking (Fig. 8*B*). Moreover, we calculated the RMS fluctuation of each amino acid residue in the *Mt*LPMO9L–CBM–cellulose–H_2_O_2_ complex during the simulation. As expected, residues in the linker region exhibited relatively high flexibility (Fig. 8*C*). Additionally, the RMSD of the CD (averaging 1.71 ± 0.01 nm) and CBM (averaging 0.81 ± 0.01 nm) concerning their average structures in all simulation runs was calculated (Fig. 8*D*). Their relatively tight distributions indicate the stable binding of the two modules to the cellulose surface.

**Table 1:** Module-cellulose interaction energies

Complexes	Free energy interaction (kJ mol^−1^)						
	Independent runs	CD-cellulose			CBM-cellulose		
		Coul-SR	LJ-SR	Sum	Coul-SR	LJ-SR	Sum
*Mt*LPMO9L-cellulose	Run1	−140.80 ± 58.44	−211.90 ± 20.08	−352.63 ± 61.79	-	-	-
	Run2	−44.56 ± 51.11	−235.50 ± 32.57	−280.09 ± 60.60	-	-	-
	Run3	−106.90 ± 64.33	−182.80 ± 29.51	−289.71 ± 70.78	-	-	-
*Mt*LPMO9L-CBM-cellulose	Run1	−145.38 ± 72.68	−182.08 ± 29.22	−327.46 ± 78.33	−101.63 ± 52.36	−173.32 ± 18.86	−274.95 ± 55.65
	Run2	−62.02 ± 53.05	−159.63 ± 25.14	−221.65 ± 57.70	−78.08 ± 69.18	−197.95 ± 37.69	−276.03 ± 78.78
	Run3	−70.61 ± 54.96	−190.71 ± 26.72	−261.32 ± 61.11	−104.09 ± 75.91	−170.03 ± 19.22	−274.12 ± 78.30

Note: Number of frames for the calculation is 50,001.

Coul-SR, columbic short-range electrostatic; LJ-SR, Lennard–Jones short-range potential; NA, not applicable.

**Figure 9:**
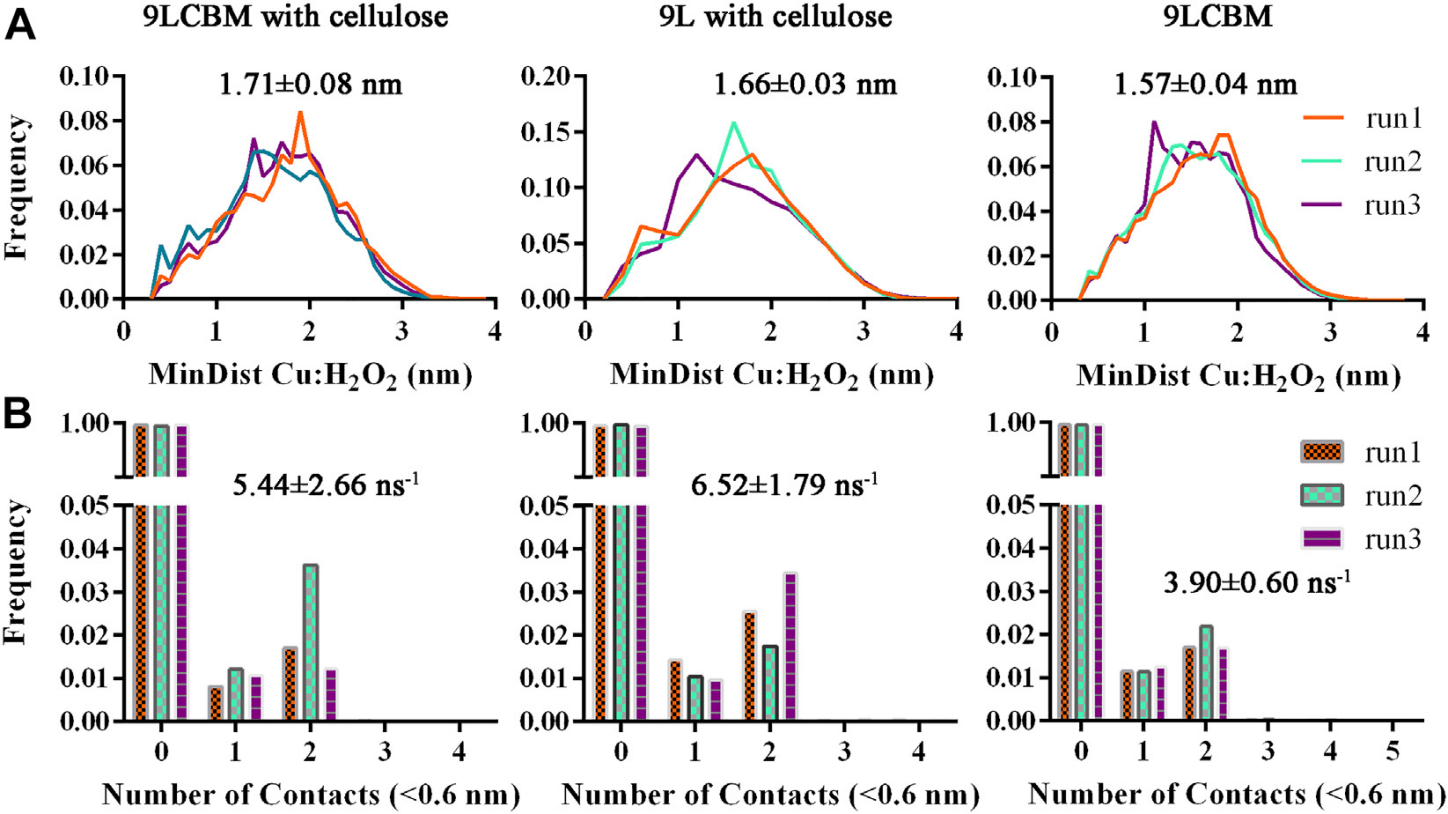
**Statistical analysis of contacts between H**_**2**_**O**_**2**_**molecules and Cu**^**2+**^**.***A*, the distribution of the minimum distance between H_2_O_2_ and Cu^2+^ in all MD runs of the three complex systems. The average distance of the three parallel runs for each complex was indicated. *B*, the number of contacts smaller than 0.6 nm between H_2_O_2_ and Cu^2+^ in each frame for all MD runs. The average frequency of the three parallel runs for each complex was indicated. The number of frames for the calculation in each run is 50,001. MD, molecular dynamics.

**Figure 10:**
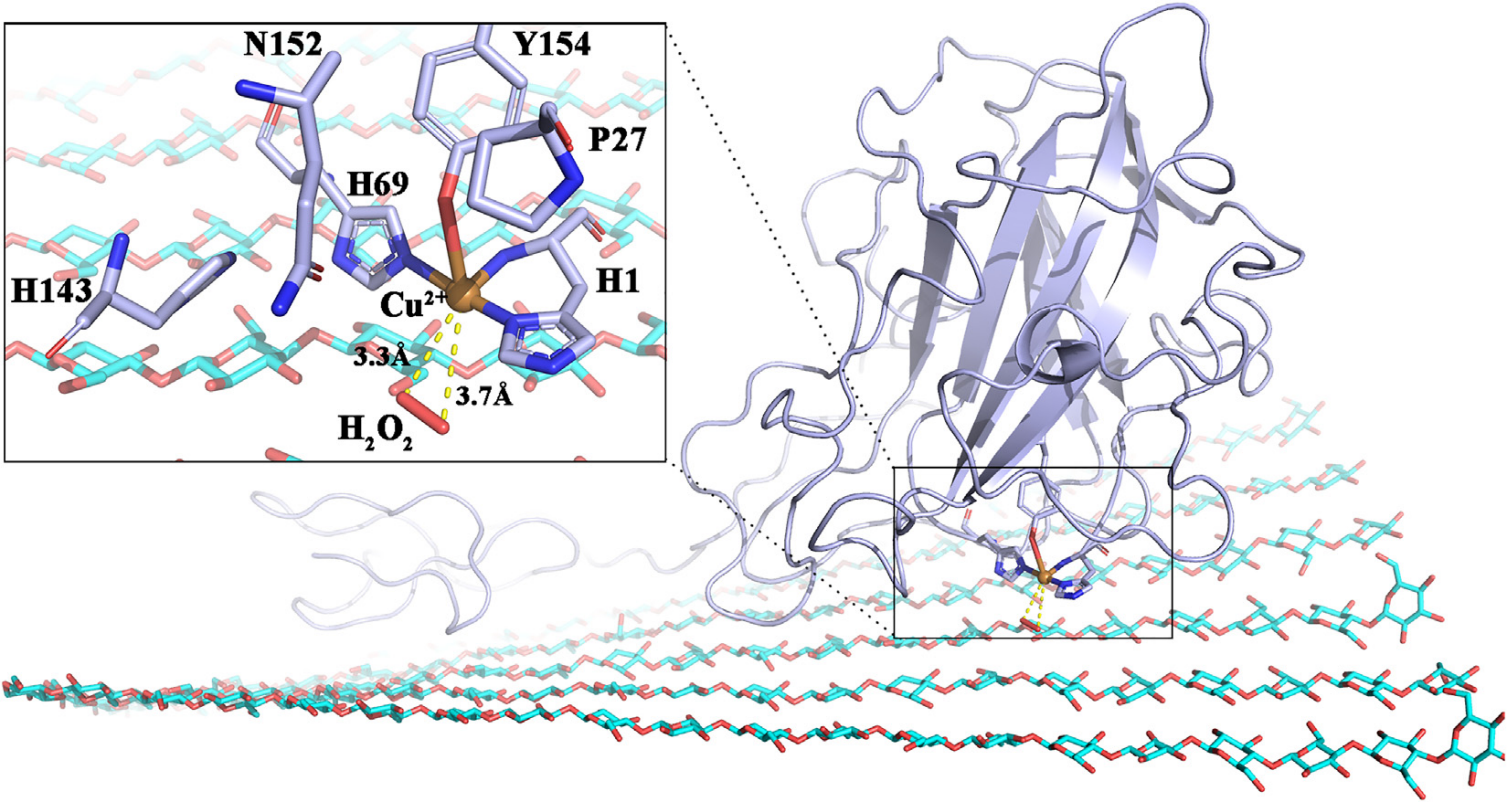
**Interaction between H**_**2**_**O**_**2**_**and *Mt*LPMO9L-CBM catalytic center residues at the closest distance in the presence of cellulose.** The residues forming the interaction cavity were labeled. The closest distance between two O atoms of H_2_O_2_ and Cu^2+^ was labeled and shown in *dashed line*. CBM, carbohydrate-binding module; LPMO, lytic polysaccharide monooxygenase.

Based on the random initial distribution of H_2_O_2_ molecules, we assessed the minimum distance from H_2_O_2_ to the enzyme copper active center in all three systems using the “mindist” function in GROMACS (Fig. 9*A*). The results showed that *Mt*LPMO9L-CBM-H_2_O_2_ exhibited the lowest average “mindist” value (1.57 ± 0.04 nm). In contrast, the “mindist” value for *Mt*LPMO9L-cellulose-H_2_O_2_ (1.66 ± 0.03 nm) and *Mt*LPMO9L-CBM-cellulose-H_2_O_2_ (1.71 ± 0.08 nm) were comparable but larger than that of *Mt*LPMO9L-CBM-H_2_O_2_ (*p* = 0.001, two-tailed *t* test). The presence of cellulose impedes H_2_O_2_ access to the LPMO active center, which is the primary reason for the lowest minimum distance value of H_2_O_2_ to the copper active center in the absence of substrate. However, what surprised us was that this value did not significantly differ from the minimum distance value of H_2_O_2_ to the copper active center when substrate was present. To provide further insight into this result, we evaluated the frequency of contact (<0.6 nm) between the active center Cu^2+^ and H_2_O_2_ molecules in the three systems. The results indicated that in the absence of substrate, the contact frequency between Cu^2+^ and H_2_O_2_ molecules in the *Mt*LPMO9L-CBM-H_2_O_2_ system was 3.90 ± 0.60 ns^−1^ (Fig. 9*B*). In contrast, in the presence of substrate, both the *Mt*LPMO9L–cellulose–H_2_O_2_ complex and *Mt*LPMO9L–CBM–cellulose-H_2_O_2_ complex exhibited increased frequencies of 6.52 ± 1.79 and 5.44 ± 2.66, respectively. In the MD trajectory of *Mt*LPMO9L-CBM–cellulose–H_2_O_2_, we observed H_2_O_2_ molecules trapped near the Cu^2+^ center through interacting with the open cavity formed by residues H1, P27, N152, and H143 (Fig. 10 and Movie S1). Indeed, it is conceivable that in the absence of substrate, H_2_O_2_ contacts the copper active center randomly, while the presence of substrate hinders this randomness, making it easier for some H_2_O_2_ molecules to be trapped near the enzyme's active center, resulting in an increased contact frequency.

Moreover, during the MD trajectory of *Mt*LPMO9L-CBM-cellulose-H_2_O_2_, we observed a slight deflection of the CD of *Mt*LPMO9L-CBM, which appeared to be influenced by the pulling forces exerted by the linker and CBM. This observation indicates a potential influence of the linker and CBM on the interaction between the CD and cellulose, which could also account for their impact on the contact between the copper active center and H_2_O_2_.

## Discussion

The role of CBMs appended to GHs has been extensively studied (1). It is widely acknowledged that CBMs can have significant effects on the substrate binding affinity, enzymatic activity, stability, and secretion levels (44, 45). However, despite some studies investigating the role of CBMs in LPMO enzymatic activity (2, 25, 46), paradoxical effects have been unexpectedly observed in certain cases. For instance, Crouch *et al*. showed that CBMs can both enhance and inhibit LPMO activity (31). Therefore, gaining a deeper understanding of the function of CBMs in LPMO catalysis is essential.

Our comparative functional characterization of single-domain enzymes and double-domain enzymes with CBM reveals a unique complexity for LPMOs, which is related to the multiple impacts of substrate affinity and binding on LPMO performance, as described above. These complexities are highly important and must be considered when interpreting existing functional data regarding the effect of CBM on LPMO efficiency, as well as in planning new studies regarding their role.

Functional studies at different substrate concentrations show that CBMs can affect the catalytic function of LPMOs, which is largely promoted by the anchoring effect due to CBM’s ability of binding to internal positions on the substrate surface, facilitating multiple cleavages on the substrate. Multiple cleavages on the cellulose surface increases the efficiency of releasing sufficient soluble products for detection by HPAEC. This finding is consistent with previous studies on *Pa*LPMO9H and its CBM truncation (46), which showed that the CBM domain has a significant role in the enzyme activity due to its strong interaction with the substrate (37, 47). Notably, the dependency of product formation on substrate concentration differs between the single-domain LPMO and dual-domain LPMO (37). The single-domain LPMO is more susceptible to inactivation at lower substrate concentrations, while the presence of CBM is crucial for prolonged activity. Although high substrate concentration can compensate for the weak substrate binding ability of single-domain LPMOs (41), the oxidative damage caused by unproductive H_2_O_2_ cannot be avoided. H_2_O_2_ is a common cosubstrate that can both promote and inhibit LPMO activity (48). Oxidative damage to the catalytic center of free LPMOs caused by excess H_2_O_2_ leads to nonproductive and potentially destructive LPMO reactions (38). Bissaro *et al*. have also proposed that LPMO reaction stability depends on enhanced enzyme activity and low H_2_O_2_ concentration at the active center (29).

To gain further insight into the important role of CBMs in LPMO catalysis, we conducted MD simulations in different systems that incorporated LPMOs, randomly placed H_2_O_2_ molecules, and cellulose. Due to the limitations in current computing power and MD simulation capabilities, we were unable to model the binding and dissociation of such a complex system in an absolutely unbiased manner to assess the true equilibrium effects of CBMs on different LPMOs in this study. Furthermore, an important constraint of using the CHARMM36 force field and most other traditional methods for macromolecule MD simulations is the inability to accurately simulate the true states of Cu^2+^ ions and H_2_O_2_. Based on this, we manually positioned the two domains of LPMO randomly on the cellulose surface, to the best of our ability within the constraints of the simulation, provided a sufficiently large time and spatial scale. This allowed us to capture, to some extent, their dynamic interactions. As expected, the results show that CBMs provide additional binding sites for LPMOs to anchor themselves onto the cellulose. The linker connecting the CBM and CD serves as a flexible spacer that maintains the distance between them while allowing them to move independently (37). In consequence, additional anchor point provided by CBM (*Mt*LPMO9L-CBM) enhanced the yield of soluble oxidized products. Moreover, as shown by the MD simulation results, the presence of cellulose significantly hindered H_2_O_2_ molecules in the solvent from randomly accessing the catalytic center. We hypothesize that this process may effectively hinder nonproductive damage to the enzyme's active center by H_2_O_2_. Furthermore, the meaningful contact between H_2_O_2_ and the catalytic center can be modulated by CBM when cellulose is present. When initiated with the same reductant concentrations, the net concentration of H_2_O_2_ in the reaction mixture of *Mt*LPMO9L-CBM system was significantly lower than that in the *Mt*LPMO9L system. The fusion of CBM effectively promotes productive binding between LPMO and the substrate while efficiently suppressing the nonproductive oxidative enzyme activity that generates excess H_2_O_2_. This ultimately results in higher catalytic efficiency and superior H_2_O_2_ utilization of *Mt*LPMO9L-CBM than the single-domain *Mt*LPMO9L.

LPMOs are a crucial component of current commercial cellulolytic enzyme cocktails (49) but their autocatalytic inactivation presents a major challenge at the industrial scale. Our study shows that the performance of LPMOs can be enhanced by CBM engineering. This is important for the development of efficient and sustainable industrial processes, such as biomass conversion and biorefinery, where LPMOs play a key role in breaking down lignocellulose. The increased H_2_O_2_ tolerance of LPMOs engineered with CBM will allow for more efficient and cost-effective use of H_2_O_2_ in these processes, reducing the need for expensive and environmentally harmful alternatives. This research thus represents an important step toward the development of more sustainable and efficient industrial processes, with significant economic and environmental benefits.

In conclusion, we used modular engineering to separately remove and fuse CBM in *Mt*LPMO9s, providing insights into the function of CBM in LPMO catalysis. Our results indicate that the contribution of CBM to enzyme activity is achieved through increased substrate binding and superior utilization of H_2_O_2_. Furthermore, the presence of substrate hinders the random accession of H_2_O_2_ into the catalytic center of LPMO, thereby preventing nonproductive oxidative damage to the copper active center. The fusion of CBM affects the contact between H_2_O_2_ and the copper active centers, which could be the reason of the enhanced H_2_O_2_ tolerance. Our study highlights the critical role of CBMs in LPMO catalysis and the potential for CBM engineering to improve LPMO performance in industrial applications.

## Experimental procedures

### Construction of *Mt*LPMO9G-CD and *Mt*LPMO9G-CDL

To generate two truncated forms of *Mt*LPMO9G, we amplified the gene region of the CD (AA1–AA205) and the gene region of the CD with linker (AA1–AA250), respectively (refer to Supporting information Table S1 for primer details). Subsequently, the amplified gene regions were inserted into pPICZαA vectors to construct recombinant vectors. *Mt*LPMO9G-CD containing only the CD and *Mt*LPMO9G-CDL containing the CD with a linker were recombinantly overexpressed as secreted proteins in *P. pastoris* X-33 and purified by anion chromatography using DEAE Sepharose CL-6B column (GE Healthcare). The enzyme purification and subsequent preparation followed the steps described in our previous work (35). To predict the three-dimensional structures of *Mt*LPMO9G and *Mt*LPMO9G-CD, we used Robetta server (50) with the method of RoseTTAFold (51) based on their amino acid sequences.

### Substrate binding of *Mt*LPMO9G, *Mt*LPMO9G-CDL, and *Mt*LPMO9G-CD by affinity precipitation

The cellulose binding affinity of *Mt*LPMO9G, *Mt*LPMO9G-CDL, and *Mt*LPMO9G-CD was compared using affinity precipitation (52, 53). Specifically, 10 mg mL^−1^ PASC was added to a 50 mM NH_4_Ac buffer (pH 5) containing 0.5 mg mL^−1^ LPMOs. The reaction was incubated at room temperature for 24 h with agitation at 1000 rpm using an Eppendorf Thermomixer. A control group without PASC was also included. After centrifugation, the supernatant was transferred to new tubes and the PASC pellet was washed with 50 mM NH_4_Ac buffer (pH 5) to remove unbound enzyme. The corresponding enzyme content in the supernatant, washing solution, and precipitate was analyzed by SDS-PAGE.

### Activity and product analysis of *Mt*LPMO9G, *Mt*LPMO9G-CDL, and *Mt*LPMO9G-CD on cellulose

The activity of *Mt*LPMO9G, *Mt*LPMO9G-CDL, and *Mt*LPMO9G-CD was evaluated using PASC as the substrate. The reaction mixture contained 50 mM NH_4_Ac (pH 5), 1 μM LPMO, 1 mM AscA, and 4 mg mL^−1^ PASC, and the reaction proceeded for 24 h at 45 °C with continuous shaking at 1000 rpm in an Eppendorf Thermomixer. The resulting products were analyzed by HPAEC using an ICS3000 system equipped with a pulsed amperometric detector (Thermo Fisher Scientific) and an analytical CarboPac PA-100 (4 × 250) as previously described (33, 34). C1-oxidized cello-oligosaccharide standards for the HPAEC-pulsed amperometric detector were prepared by oxidizing pure cello-oligosaccharides (Megazyme) with iodine as described earlier for the preparation of cellobionic acid (54). Additionally, the products were hydrolyzed by cellobiohydrolase Ⅰ(CBH Ⅰ) (Megazyme) and quantitatively analyzed by HPAEC.

### Construction of *Mt*LPMO9L-CBM

Sequence alignment of *Mt*LPMO9G and *Mt*LPMO9L was performed by ClustalW software (http://www.genome.jp/tools-bin/clustalw) (55), and a more intuitive alignment was presented by ESPript 3.0 (http://espript.ibcp.fr/ESPript/cgi-bin/ESPript.cgi) (56). Next, the non-CD module (AA206–AA284) of *Mt*LPMO9G was fused to the C terminus of *Mt*LPMO9L to construct a fusion form (*Mt*LPMO9L-CBM) (refer to Supporting information Table S2 for primer details). The recombinant enzymes, *Mt*LPMO9L and *Mt*LPMO9L-CBM, were recombinantly overexpressed as secreted proteins in *P. pastoris* X-33 and purified by anion chromatography using Q-Sepharose fast-flow (GE Healthcare) (57). The Purified enzymes were saturated with CuSO_4_ and desalted using an Amicon ultracentrifugation device equipped with a 10 kDa cut-off membrane, as previously described by R. Kont *et al*. (40). Furthermore, the three-dimensional structures of *Mt*LPMO9L and *Mt*LPMO9L-CBM were predicted based on their amino acid sequences using Robetta server (50) with the method of RoseTTAFold (51).

### Substrate binding of *Mt*LPMO9L and *Mt*LPMO9L-CBM by affinity precipitation and pull-down assay

The cellulose binding affinity of *Mt*LPMO9L and *Mt*LPMO9L-CBM was compared using affinity precipitation as described in section 4.2. Next, we further analyzed the binding affinity by pull-down assay (3, 33). PASC, derived from Avicel with an average degree of polymerization of about 200 (58), was employed at varying concentrations (0–120 μM). These concentrations were incubated with 1.5 μM LPMOs under agitation at 1000 rpm for 2 h. Subsequently, the concentration of unbound enzyme in the supernatant was detected using a Quick Start Bradford reagent kit (Bio-Rad). Each assay was performed in triplicate. The binding isotherms were fitted to the one-point binding equation (Equation 1) using nonlinear regression in Prism 7 software (https://www.graphpad.com/scientific-software/prism, GraphPad, Inc) to determine the equilibrium *K*_*d*_ and substrate-binding capacity (Bmax) (33). [B] represents the concentration of bound protein, and [F] represents the concentration of free protein.

(1) $$\left[B\right]=\frac{B_{\max}\;\times \;\left[F\right]}{K_{d}\;+\;\left[F\right]}$$

### Activity assay and time course analysis of *Mt*LPMO9L and *Mt*LPMO9L-CBM

The activity of *Mt*LPMO9L and *Mt*LPMO9L-CBM was evaluated using a standard LPMO reaction mixture consisting of 1 μM LPMO, 1 mM AscA, and 4 mg mL^-1^ PASC. The reaction mixture was incubated in 50 mM NH_4_Ac (pH 5) at 45 °C for 24 h with continuous shaking at 1000 rpm in an Eppendorf Thermomixer. The products were analyzed by high performance anion exchange chromatography as described in section 4.3. Time-course experiments were also performed with standard LPMO reactions, which were performed in triplicate and lasted for 72 h. Prior to high performance anion exchange chromatography analysis, the products were hydrolyzed by CBH Ⅰ (Megazyme). Additionally, the concentration of H_2_O_2_ during LPMO reactions was determined using the Fluorometric Hydrogen Peroxide Assay Kit (Sigma), following a modified HRP/Amplex Red assay protocol (59) originally proposed by Kittl *et al*. (60).

### Thermal stability determination of *Mt*LPMO9L and *Mt*LPMO9L-CBM by DSC

The thermal stability of *Mt*LPMO9L and *Mt*LPMO9L-CBM was assessed using a TA Instrument model DSC Q100. The instrument was calibrated using indium for temperature calibration. The samples were sealed in aluminum sample pans and heated at a rate of 1 °C min^-1^ from 30 °C to 85 °C. Baseline correction was performed using buffer, and data analysis was conducted with the NanoAnalyze program. The sample baseline correction was performed using a spline function. The data points were fitted to a nontwo-state thermal unfolding model after subtraction of buffer baselines and normalization for protein concentration.

### The H_2_O_2_ tolerance of *Mt*LPMO9L and *Mt*LPMO9L-CBM

The reaction mixture comprised of 4 mg mL^-1^ PASC, 1 μM LPMO, 1 mM AscA, and varying concentrations of H_2_O_2_ (0, 50, 100, 200, and 400 μM), all dissolved in 50 mM NH_4_Ac buffer (pH 5). Each group was performed in triplicate. The reaction was performed in a 2 ml tube at 45 °C with shaking at 1000 rpm in an Eppendorf Thermomixer for 48 h. After the sample was digested with CBH Ⅰ (Megazyme), the products were quantitatively analyzed by HPAEC.

### MD simulations of *Mt*LPMO9L-CBM binging to cellulose

The initial conformation of *Mt*LPMO9L-CBM was generated using the Robetta server (50) with the method of RoseTTAFold (51). The Iβ crystallite cellulose model was constructed using the Cellulose-Builder server (61). The generated parallelepiped cellulose crystal model comprised 3, 8, and 10 unit cells in the *a*, *b*, and *c* crystallographic directions, respectively. With the combination of the enzyme, cellulose, and H_2_O_2_ molecule, three different complexes were established. The first complex, *Mt*LPMO9L–cellulose–H_2_O_2_, positioned *Mt*LPMO9L on the cellulose model surface based on the previously described method (33). The second complex, *Mt*LPMO9L–CBM–cellulose–H_2_O_2_, determined the binding position of its CD on cellulose using the same method mentioned above. As for the CBM, its initial position on the cellulose surface was established by manually placing the module with a distance within the hydrophobic interaction range (3.3–4.0 Å) using the coot software (https://www2.mrc-lmb.cam.ac.uk/personal/pemsley/coot/) (62). The linker segment of the chimera protein was adjusted and repaired using the Regularize Zone function in Coot. It was then left in a randomized conformation, situated at a considerable distance from the cellulose surface. The third complex, *Mt*LPMO9L–CBM–H_2_O_2_, did not contain a cellulose substrate. In all three complex systems, the ratio of enzyme: H_2_O_2_ was set to 1:100. Additionally, the positions of all H_2_O_2_ molecules in all systems were randomly placed by the “insert-molecules” function of the GROMACS program (63).

The input files for GROMACS MD simulation were generated using the solution builder module of the CHARMM-GUI input generator (http://www.charmm-gui.org) (64). The default CHARMM36 force field was applied in the wizard. During this process, the topologies of cellulose chains, Cu^2+^, and H_2_O_2_ were automatically generated. All complexes were contained in cubic water boxes with similar size and solvated with the transferable intermolecular potential by three points (TIP3P) water model (65). To maintain system neutrality, the appropriate number of Na^+^ or Cl^-^ ions was added using the Monte-Carlo method. The input option was configured for GROMACS, and the temperatures of all systems were set to 303.15 K. Subsequently, initial MD simulations were conducted using GROMACS v2021.3, including energy minimization, temperature equilibration (NVT), pressure equilibration (NPT), and production simulation. Following these initial steps, 500 ns simulations were performed for all three systems to explore the dynamic distribution of H_2_O_2_ atoms in the presence or absence of cellulose, as well as the interaction between CBM and cellulose. To increase the statistical significance of the simulations, three replicates were performed for each system. These replicates initiated from the same coordinates but with different initial velocities, generated according to a Maxwell-Boltzmann distribution. The output trajectory of simulations was centered and analyzed by the built-in functions of the GROMACS (https://www.gromacs.org/) software. The trajectory visualizing inspections were performed by VMD (https://www.ks.uiuc.edu/Research/vmd/) software (66).

## Data availability

The data that support the findings of this study are available from the corresponding author upon reasonable request.

## Supporting information

This article contains supporting information.

## Conflict of interest

The authors declare that they have no conflicts of interest with the contents of this article.
