# Supplementary material for: Carbohydrate-binding modules enhance H2O2 tolerance by promoting lytic polysaccharide monooxygenase active site H2O2 consumption
Source: J Biol Chem. 2023 Dec 18;300(1):105573. doi: 10.1016/j.jbc.2023.105573 (PMC10825053; doi:10.1016/j.jbc.2023.105573)
Supplement: Supporting Information Movie S1 [file mmc2.docx]

# Movie S1. A concise video presenting the dynamic interaction between H_2_O_2_ molecules and the catalytic center of the *Mt*LPMO9L-CBM-cellulose complex. During the simulation, the distance changes between copper and the H_2_O_2_ molecule with the minimum distance to copper are marked. Key residues involved in cellulose interactions, along with those participating in the primary and secondary coordination spheres of copper, are depicted in stick representations.
