# Supplementary material for: Carbohydrate-binding modules enhance H2O2 tolerance by promoting lytic polysaccharide monooxygenase active site H2O2 consumption
Source: J Biol Chem. 2023 Dec 18;300(1):105573. doi: 10.1016/j.jbc.2023.105573 (PMC10825053; doi:10.1016/j.jbc.2023.105573)
Supplement: Supporting Information [file mmc3.docx]

Supplementary Information for

**Carbohydrate-Binding Modules Enhance H_2_O_2_ Tolerance by Promoting Lytic Polysaccharide Monooxygenase Active Site H_2_O_2_ Consumption**

Wa Gao^1,2^, Tang Li^1^, Haichuan Zhou^1^, Jiu Ju^1^, Heng Yin^1,2,*^

*^1^ Dalian Engineering Research Center for Carbohydrate Agricultural Preparations,* *Dalian Technology Innovation Center for Green Agriculture, Liaoning Provincial Key Laboratory of Carbohydrates, Dalian Institute of Chemical Physics, Chinese Academy of Sciences, Dalian, 116023, China*

*^2^ University of Chinese Academy of Sciences, Beijing, 100049, China*

*Corresponding author. E-mail address: yinheng@dicp.ac.cn


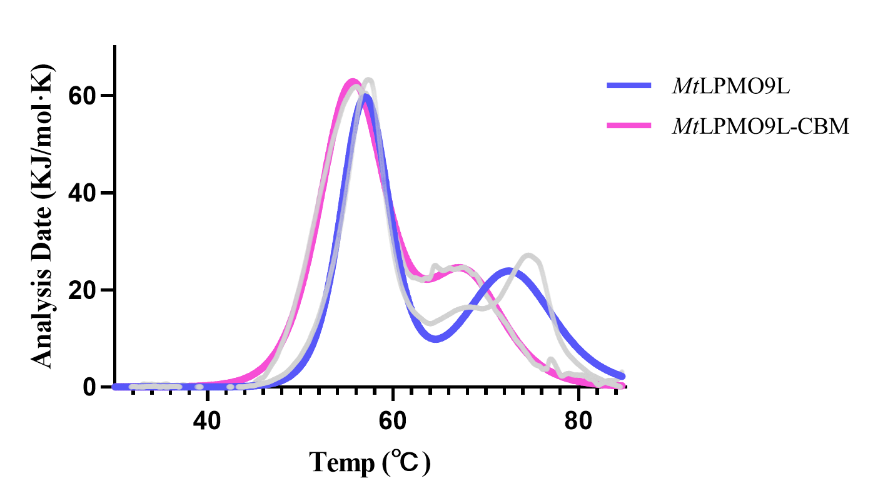


Figure S1. Thermal unfolding of *Mt*LPMO9L and *Mt*LPMO9L-CBM. The melting temperatures (*T_m_*) were determined using differential scanning calorimetry (DSC) by heating the enzymes at a rate of 1 °C min^-1^ from 30 °C to 85 °C. The original data is represented by the gray solid line, while the fitting curves for *Mt*LPMO9L and *Mt*LPMO9L-CBM are shown by blue and pink solid line, respectively.


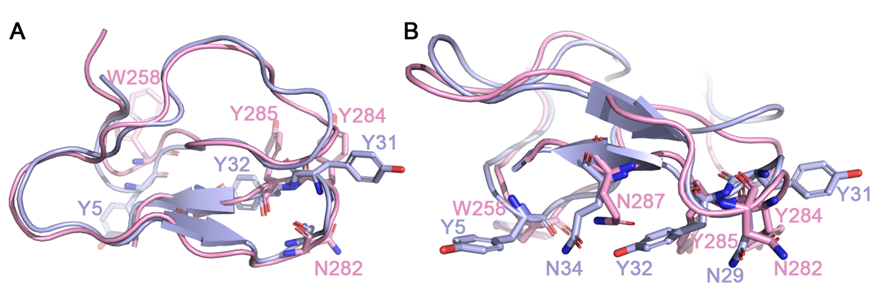


Figure S2. Top (A) and side (B) view of the superimposed structures of CBM from *Mt*LPMO9G (pink) and *Tr*Cel7A (light blue).

Table S1. Primers used for the construction of *Mt*LPMO9G-CDL and *Mt*LPMO9G-CD, including sequences of sense and antisense primers.

|  | Primer pair, sense and antisense (5’→3’) |  |
| --- | --- | --- |
| *Mt*LPMO9G-CDL | GACCTCGAGAAAAGACACTACATCTTTCAGCAGC | |
|  | CTTGCGGCCGCCTAGACGGTGCAGCTGGAGG | |
| *Mt*LPMO9G-CD | GACCTCGAGAAAAGACACTACATCTTTCAGCAGC | |
|  | CTTGCGGCCGCCTAGCAGGTGAAGACGGCTGG | |

Table S2. Primers for the construction of *Mt*LPMO9L-CBM, including sequences of sense and antisense primers.

|  | Primer pair, sense and antisense (5’→3’) |  |
| --- | --- | --- |
| *Mt*LPMO9G-CDL | CTACACTCCCGCTGGTCCCCCCGTCGACACCTGCAACGGTAGCGGCGGCAACAAC | |
|  | CTGAGGAACAGTCATGTCTAAGGCTACAAACTCACAAGCACTGGCTGTAGTAGTCG | |

Table S3. Substrate binding parameters of *Mt*LPMO9L and *Mt*LPMO9L-CBM, including dissociation constant (*K_d_*) and maximum binding capacity (*B_max_*). The uncertainties presented are expressed as standard errors.

|  | *Mt*LPMO9L | *Mt*LPMO9L-CBM |
| --- | --- | --- |
| *K_d_* (μΜ) | 13.1±8.5 | 2.1±0.2 |
| *B_max_* (μΜ) | 0.41±0.07 | 1.49±0.02 |
